# *Staphylococcus capitis* Central-Line-Associated Bloodstream Infections in the Neonatal Intensive Care Unit: A Single-Center, Four-Year Experience in Central-Line Management during Sepsis Treatment

**DOI:** 10.3390/pathogens13030234

**Published:** 2024-03-07

**Authors:** Anna Sala, Valentina Pivetti, Alessandra Vittorini, Claudia Viggiano, Francesca Castoldi, Valentina Fabiano, Gianluca Lista, Francesco Cavigioli

**Affiliations:** 1 Department of Neonatology and Neonatal Intensive Care Unit, V. Buzzi Children’s Hospital, 20154 Milan, Italy; anna.sala1@unimi.it (A.S.); valentina.pivetti@asst-fbf-sacco.it (V.P.); alessandra.vittorini@unimi.it (A.V.); francesca.castoldi@asst-fbf-sacco.it (F.C.); gianluca.lista@asst-fbf-sacco.it (G.L.); 2 Department of Biomedical and Clinical Sciences, University of Milan, 20157 Milan, Italy; valentina.fabiano@unimi.it; 3 Department of Pediatrics, V. Buzzi Children’s Hospital, 20154 Milan, Italy; 4 Department of Neonatology and Neonatal Intensive Care Unit, Macedonio Melloni Hospital, 20129 Milan, Italy; claudia.viggiano@asst-fbf-sacco.it

**Keywords:** LOS, CLABSI, CoNS, CVC, infants, *S. capitis*

## Abstract

Coagulase-negative staphylococci (CoNS) are reportedly responsible for 50–60% of bloodstream infections in very preterm (<1500 g) infants in neonatal intensive care units (NICUs). *Staphylococcus capitis* is an increasingly prevalent pathogen in the neonatal setting, frequently causing central-line-associated bloodstream infections (CLABSIs) that can be difficult to eradicate. Central venous catheter (CVC) removal versus in situ treatment with CoNS CLABSIs is a controversial treatment strategy with no clear consensus. We reviewed all *S. capitis* CLABSIs in our NICU between 2019 and 2022, focusing on the role of catheter removal in eradication. Among the 25 patients, 17 CVCs were removed after diagnosis, leading to a 76.5% eradication rate in this group. Three infants had a persistently positive blood culture after CVC substitution. A new catheter was then inserted after a 48 h washout period, resulting in resolution of the infection. Only two of the eight patients (25%) who retained their catheter after diagnosis achieved infection eradication with antibiotic therapy alone. When feasible, catheter removal seems to be the most effective strategy for eradicating *S. capitis* CLABSIs, sometimes even requiring a 48 h washout period before reinsertion. Further studies on this topic are needed to better standardize the management of this type of infection.

## 1. Introduction

Bacterial infections and sepsis are frequent complications in the neonatal intensive care unit (NICU) due to the immune immaturity of preterm neonates and the frequent need for the use of invasive devices such as endotracheal tubes, drainages, or central venous catheters [1]. The signs and symptoms of sepsis that become evident during the first 72 h of life indicate early-onset sepsis (EOS), which is typically caused by the vertical transmission of bacteria colonizing the lower genital tract of the mother via contaminated amniotic fluid or infection of the baby during vaginal delivery [2]. Sepsis that occurs after 72 h of life is called late-onset sepsis (LOS) and is a common event, especially in very-low-birthweight (VLBW) infants, affecting approximately 20 to 30% of patients at least once during hospitalization [3] and being a significant cause of morbidity and mortality in this population [4,5]. Vertical transmission of maternal pathogens (e.g., transmission through the mother’s contaminated milk) is a possible etiology even in this kind of sepsis, although it is rarely implied [6]. LOS usually derives from horizontal transmission of environmental pathogens, occurring despite strict hygiene measures and prevention strategies [7]. Healthcare-associated infections (HAIs) represent the most prevalent cause of LOS in the NICU and may be caused by the longer dwell time of invasive devices and the repeated need for invasive procedures in this vulnerable population [3]. The temporal peak of the LOS incidence occurs between the 10th and 22nd days of life [8]. LOS in the NICU can be caused by several different pathogens, among which skin commensal coagulase-negative staphylococci (CoNS) are the most commonly involved, accounting for at least half of all cases; other possibly implicated pathogens are *Staphylococcus aureus* and gram-negative bacteria such as *Escherichia coli*, *Klebsiella pneumoniae*, and *Acinetobacter baumannii* [5,9,10,11,12,13,14,15].

In the adult population, the isolation of CoNS from blood culture is often considered a simple contamination, and in the NICU setting, the clinical significance of this finding has become increasingly evident over the years [16,17]. Over the last few decades, *Staphylococcus epidermidis* has emerged as the most prevalent CoNS causing LOS in preterm neonates [9], but during the last few years, several studies have reported the isolation of different species of CoNS, including *Staphylococcus capitis,* which has become increasingly common in preterm infants with LOS [18,19].

In particular, a specific clone of methicillin-resistant *Staphylococcus capitis* (pulsotype NRCS-A) has been isolated in hospitals from different countries worldwide (Australia, Belgium, France, and the UK), showing particular specificity for the NICU environment and reduced susceptibility to vancomycin, one of the first-line empiric antibiotics used in LOS in some countries [20,21]. The reason for this widespread diffusion and persistence in such a specific setting has been investigated, and possible reservoirs have been identified in body care oil bottles [22], stethoscopes, and neonatal incubators, which were found to be colonized despite being repeatedly sanitized [23]. Persistence on surfaces might be linked to decreased susceptibility to some disinfectants [23,24]. Screening for caregiver colonization has also been performed, revealing no chronic carriage of *S. capitis* [25] or isolation of a different clone than NRCS-A [23].

All *S. capitis* strains isolated in NICUs usually exhibit multidrug resistance patterns, especially those involving beta-lactams and aminoglycosides, which are widely used in this setting [20]. Resistance or heteroresistance to vancomycin has also been reported in several studies [26,27], even though these findings have not been reported by all authors [23,28] and have, in some cases, been described as a specific characteristic of only the NRCS-A clone [29].

For all sepsis caused by CoNS, central venous catheters (CVCs) are often identified as the source of infection in *S. capitis* LOS, supported by the ability of this pathogen to form a biofilm [30,31,32]. CoNS are the most common pathogens involved in central-line-associated bloodstream infections (CLABSIs) [33], defined by the Center for Disease Control and Prevention (CDC) as a primary laboratory-confirmed bloodstream infection (BSI) in a patient who had a central line within the period 48 h before the development of the BSI that is not related to an infection at another site [34]. This definition is mainly used for surveillance purposes and differs from the stricter definition of catheter-related bloodstream infections (CRBSIs), which require the presence of clinical symptoms, along with a positive blood culture from a peripheral vein. In addition, the same organism must be detected from the catheter segment culture using any of the following methods: (i) semiquantitative or (ii) quantitative catheter culture with a positive result or (iii) simultaneous quantitative cultures, with differential times to positivity of the catheter vs. the peripheral blood [35]. Central line removal in cases of CLABSIs caused by CoNS is controversial, and a conservative management strategy is sometimes suggested, considering the relevance of having secure vascular access for parenteral nutrition and therapy in preterm neonates [36,37].

The objective of our study was to describe and analyze *S. capitis* CLABSIs that occurred in our NICU from the first isolation of the pathogen in 2019 until 2022, focusing on the timing of central line removal and how it affects the outcome of the infection.

## 2. Materials and Methods

We performed a retrospective observational single-center study. We revised the existing database of CLABSIs in the Buzzi Children’s Hospital NICU, which included data collected in 2016, and selected patients who had *S. capitis* infection. Demographic and clinical data were collected from clinical records, including details on the type of central line used, date of insertion and date of removal. Blood culture data (time to positivity, antibiogram, minimum inhibitory concentration) were obtained from the electronic patient record system. Blood cultures were analyzed in the Luigi Sacco Hospital microbiology laboratory as per regular practice, with antibiograms drafted according to the most recent EUCAST breakpoint tables.

Descriptive analyses were performed using Stata (version 17.0; StataCorp LLC, College Station, TX, USA). All the data are presented as means, medians, and percentages.

The study was conducted in accordance with the Declaration of Helsinki and approved by the Ethics Committee of the coordinating center in Milan (protocol number 30581/2023 of 3 July 2023).

## 3. Results

Based on the preexisting CLABSI database, we found that the first catheter-related infection caused by *S. capitis* in our NICU occurred in 2019; then, we revised the subsequent 4-year period (2019–2022), during which we recorded a total of 78 CLABSIs, of which 54 were caused by CoNS with 25 *S. capitis*-related CLABSIs (Table 1). The percentage of *S. capitis* infections out of the total number of CLABSIs ranged annually from 20.8% to 42.9%. During this period, the total number of admissions to our unit was 461 (43 VLBW) in 2019, 443 (42 VLBW) in 2020, 433 (62 VLBW) in 2021, and 417 (53 VLBW) in 2022.

Within the total population of 25 newborns with *S. capitis* CLABSIs (Table 2), 5 were late preterm or term neonates who needed a central catheter due to different clinical conditions requiring gastrointestinal surgical intervention and a subsequent fasting period: an anorectal malformation, a VACTERL (vertebral defects, anal atresia, cardiac defects, tracheo-esophageal fistula, renal anomalies, and limb abnormalities) association, a gastroschisis, a meconium ileus in a patient with cystic fibrosis, and a congenital diaphragmatic hernia. The remaining 20 were all preterm VLBW babies necessitating prolonged insertion of a central vascular catheter for parenteral nutrition support or intravenous therapy. The comorbidities of these babies are listed in Table 2; none of them at the onset of sepsis presented a different infectious focus or pathology justifying clinical instability or high inflammatory markers, as also stated in the CLABSI’s definition.

The median gestational age of the infected neonates was 28 weeks (range 24 + 1–38 + 6 weeks), with a median birthweight of 885 g (range 491–2950 g). The mean length of hospital stay was 88.3 days. One out of 25 patients had a subsequent fatal outcome related to severe bronchopulmonary dysplasia complications and not directly related to the *S. capitis* LOS.

Most of the central catheters in place during the onset of the infection were 1 French epicutaneo-caval polyurethane catheter (22 out of 25); 2 were centrally inserted central catheters (CICCs), both in polyurethane, with diameters of 3 and 4 French, inserted in the right internal jugular vein. Only in one patient did central vascular access involve a 3.5 French umbilical venous catheter (UVC) in polyurethane (Table 3).

The median dwell time of the epicutaneo-caval catheter group was 15 days (range 6–21 days), that of the two CICCs was 11 and 14 days, and that of the UVC group was 6 days.

At the time the infection was suspected and blood culture was performed, catheters were positioned for a mean of 8.84 days.

Bloodstream infections were identified by performing blood cultures: they were drawn from both the catheter and from a peripheric vein only in 15 patients due to the widespread use of small-diameter epicutaneo-caval catheters that are not suitable for blood sampling. The other 10 patients had only peripheral blood cultures available.

### 3.1. Catheter Removal

Concerning catheter removal, after the first positive blood culture for *S. capitis*, 17 out of 25 patients (68%) had their central catheter removed, with a mean time of 3.24 days from the day the blood culture was performed to the day of removal. Of these 17 patients, 13 (76.5%) had a subsequent negative blood culture, which was performed a mean of 5.15 days after removal. In 11 of the 13 patients, the catheter was immediately replaced with a new catheter, while in 2 patients, the catheter was not inserted because it was no longer necessary.

Conversely, 2 out of 17 patients had a positive blood culture after removal: they were left for 2 days without a central catheter as a “washout” period. A new central catheter was then positioned, and both blood cultures, performed 3 and 7 days after the previous one, were negative.

Of these 17 patients, 1 had his umbilical catheter substituted with an epicutaneo-caval catheter after blood culture positivity (6 days after placement). Four days later, a second blood culture was performed, which was still positive. Given the serious conditions of the patient requiring a stable central line, another central catheter was immediately placed. Blood cultures 24 and 48 h after positioning were positive. An attempt was then made to leave the patient for 2 days with just a peripheric catheter: blood culture drawn after 48 h was negative, and a central catheter was then safely repositioned.

The last of the 17 patients who had the catheter replaced was a surgical patient who immediately positioned a new long-term CICC and underwent a 14-day course of treatment with vancomycin, stabilizing his clinical condition without repeating a second blood culture. After 24 days, due to clinical instability, another blood culture was taken, revealing persistent positivity for *S. capitis*; the antibiogram still showed adequate sensitivity to vancomycin (Table 4). To rule out the presence of previously unidentified infectious foci, an echocardiogram and an abdominal ultrasound were performed. A second course of vancomycin was administered without removing the CICC, and a negative culture was obtained after 12 days of therapy.

On the other hand, of the eight patients (32%) who did not have the catheter removed, only two (25%) had a subsequent negative blood culture after 5 and 7 days of antimicrobial therapy. Five of them had their central catheter removed after the second positive culture: one was no longer necessary, while the other four were immediately replaced. All five blood cultures taken at a mean of 4.8 days after removal were negative.

**Figure 1:**
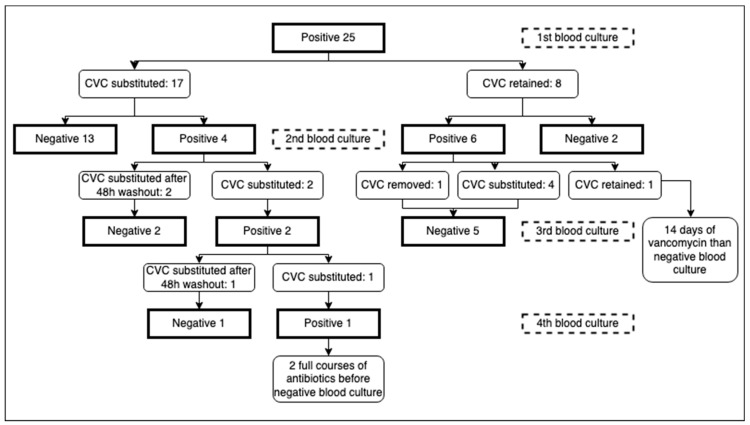
Flow diagram of blood culture execution and timing of CVC removal. CVC = central venous catheter.

The catheter was kept in place for one patient even after the second positive culture, due to his very unstable conditions: an echocardiogram and an abdominal ultrasound were performed, revealing no infectious foci. The *S. capitis* strains isolated showed adequate sensitivity to vancomycin (Table 4). After a full 14-day course of vancomycin, the last blood culture was negative (Figure 1).

### 3.2. Clinical Presentation

The clinical manifestation of LOS in our population was mainly respiratory instability (18 out of 25), represented by a sudden increase in desaturation episodes or frequent apneas and occasionally requiring a higher level of respiratory assistance (three patients required endotracheal intubation and mechanical ventilation), not explained by a specific acute pulmonary condition. Ten patients presented with fever, four of whom had both respiratory instability and hyperpyrexia. Only two patients developed hemodynamic instability with low blood pressure requiring crystalloid fluids in boluses to maintain adequate perfusion; both of them also had fever and respiratory symptoms. For one patient, clinical presentation details were not available.

### 3.3. Antimicrobial Therapy and Antibiogram

All patients started empiric parenteral antibiotic therapy immediately after blood culture with oxacillin and amikacin following our internal department protocol for the management of LOS. As soon as the antibiogram was available, each patient was switched to treatment with vancomycin via continuous parenteral infusion for 10 to 14 days. All *S. capitis* strains isolated were, in fact, oxacillin- and gentamicin-resistant but susceptible to vancomycin, with a stable minimum inhibitory concentration (MIC) of 1 for every antibiogram throughout the study period (Table 4).

## 4. Discussion

Although CLABSIs represent a common clinical situation encountered in our NICUs, there is a lack of available research regarding the early removal or retention of catheters. BSIs caused by *Staphylococcus aureus*, *enterococci*, gram-negative bacilli, and *Candida* spp. have been reported in some observational studies to require prompt CVC removal to avoid complicated or persistent sepsis [37,38,39,40,41]. The most recent Infectious Disease Society of America (IDSA) guidelines for the management of catheter-related infections also identify BSIs from *S. aureus*, fungi, and *P. aeruginosa* as situations requiring the removal of long-term catheters, while in case of the isolation of coagulase-negative staphylococci, immediate catheter removal is not suggested [35].

A 2002 survey conducted across 34 neonatal intensive care units in the United States revealed that 61% of interviewed neonatologists would not routinely remove a peripherally inserted CVC upon detecting a positive blood culture for CoNS, eventually reevaluating their choice based on patient clinical improvement or the persistence of positive cultures [42]. This work underscores the consideration given to individual patient circumstances, with healthcare providers opting for catheter removal based on clinical indicators and microbial clearance rather than a standardized protocol.

In this retrospective observational study, we found that prompt catheter removal for *S. capitis* CLABSIs resulted in sepsis resolution in 76.5% of patients, whereas a first attempt to retain the catheter was successful in 25% of patients. Seventy-five percent of patients who retained the catheter and remained positive eventually underwent catheter removal to resolve the infection; this finding is consistent with the IDSA guidelines, suggesting that in patients in whom treatment without catheter removal was attempted due to difficulty in finding alternative access, a persistent or recurrent BSI should still lead to CVC removal [35].

Few other similar retrospective studies are available. Karlowicz et al. [43] reported a success rate of 46% with catheter retention during CoNS bacteremia, which is markedly greater than our finding. This difference may be influenced by the lower retention rate found in our data: in their study, CVCs were retained for more than 3 days in approximately half of the patients (63 out of 119, 52%), while in our population, only 8 out of 25 patients (32%) maintained central access. This tendency may be justified by the growing awareness in our department regarding the widespread diffusion and persistence of *S. capitis* in the NICU setting, which has led to its emergence among other CoNS. The different success rates may also be influenced by the pathogens involved: Karlowicz’s work considered all CoNS sepsis, while we focused only on *S. capitis*, a pathogen possibly causing more protracted forms of CLABSI than other CoNS due to its described microbiological characteristics of colonization and persistence on surfaces and its heteroresistance to vancomycin [23,26].

Deshpande et al. [44] reported that the catheter clearance rate for retained catheters during CLABSIs caused by CoNS was greater than that described in our study (39% vs. 25%). This work also considered all different CoNS together; therefore, this difference might be explained by the same considerations described above.

Neither study reported a difference in mortality or length of hospital stay between the groups that had retained the CVC and those that had it removed during CoNS CLABSI [43,44]. Nevertheless, Deshpande et al. reported that CVC retention increased the duration of bacteremia and the use of systemic antibiotics [44].

In our study, three patients with persistent positive blood cultures after CVC substitution (Figure 1) were managed with a “washout” strategy, waiting for 48 h between catheter removal and reinsertion of a new catheter. It has been reported that immediate reinsertion of a CVC removed because of a CLABSI may cause persistent or recurrent infections [45] or even a higher mortality [46]; however, the washout strategy has been proven to be useful, especially for fungal infection, while for other pathogens, there is no strong evidence of efficacy [47,48]. Nevertheless, in our three patients, avoiding immediate reinsertion of the CVC led to negative blood cultures and eradication of persistent blood infection, which in those patients could not be achieved by immediately changing the central line. Implementing this strategy in newborns is inherently challenging due to the unique characteristics of this patient population: neonates frequently rely on central access for vital medications, nutrition, and other life-sustaining therapies, making temporary catheter removal a complex decision and sometimes an impossible option. The peculiarities of neonatal patients and their clinical needs can constitute an obstacle to the application of a 48 h waiting period. In many cases, these infants may not tolerate prolonged periods without central access; consequently, the practicality of this strategy must be carefully weighed against the potential risks associated with interrupting essential medical support in this particular population or switching to possibly less adequate support through a peripheric vein when feasible. However, from our experience, we believe that in selected situations, this management strategy needs to be taken into consideration to achieve the fundamental objective of sepsis eradication.

In our population, the clinical presentation was nonspecific and aligned with that commonly observed in cases of late-onset sepsis caused by coagulase-negative staphylococci; these infections are generally less severe and have a lower lethality than those induced by gram-negative pathogens [49]. Our findings are consistent with the literature, which suggests that sepsis attributed to *S. capitis* is typically associated with a milder clinical course [18].

Regarding antimicrobial therapy and resistance, in our study, all the *S. capitis* strains isolated were found to be resistant to beta-lactams and aminoglycosides, while no resistance to vancomycin was detected. This finding is consistent with what has been reported in the literature: beta-lactam and aminoglycoside resistance is induced by the selective pressure of the NICU setting in which they are widely used [50]. The absence of vancomycin resistance in the strains of *Staphylococcus capitis* identified in our department can be attributed to the fact that vancomycin is not routinely used as a first-line treatment, reducing the spread of *S. capitis* clones with heteroresistance to vancomycin, similar to what is reported in New Zealand [23].

Several limitations warrant consideration when interpreting the findings of our study. First, the retrospective observational design of our study introduces inherent biases and limits our ability to establish causal relationships. Moreover, our population size remains relatively small, potentially reducing the generalizability of our results. Additionally, due to limitations in laboratory methodologies, we were unable to ascertain the genotype of the *S. capitis* strains isolated; thus, we did not know if we were dealing with the NRCS-A clone.

## 5. Conclusions

*S. capitis* has emerged as an increasingly prevalent pathogen within our NICUs and is often associated with infections related to CVCs. Its persistence in clinical settings poses challenges due to its recalcitrance to eradication. The removal of the central catheter in such cases seems to work as a strategy to facilitate pathogen elimination, leading to the consideration of a 48 h washout period if feasible. Presently, the decision to remove the CVC after an established diagnosis of CLABSI remains a subject of controversy and is evaluated on a case-by-case basis. Further randomized controlled trials on larger populations could better substantiate the efficacy of a removal approach, possibly associated with a shorter antibiotic course, and try to establish standardized protocols for the management of these infections.

## Figures and Tables

**Table 1:** Number of CLABSIs per year, specifying the number of CoNS- and *S. capitis*-related infections. CLABSI = central-line-associated bloodstream infection, CoNS = coagulase-negative staphylococci.

	2019	2020	2021	2022	Total
Total no. of CLABSI	15	24	25	14	78
no. of CoNS CLABSI	9	18	16	11	54
no. of *S. capitis* CLABSI (% of total)	5 (33.3)	5 (20.8)	9 (36)	6 (42.9)	25

**Table 2:** Demographic characteristics of the population in the present study. M = male, F = female.

	No. tot = 25
F (%)	10 (40)
M (%)	15 (60)
Gestational age (range)	28 (24 + 1–38 + 6)
<28 weeks (%)	11 (44)
28–32 weeks (%)	9 (36)
32–35 weeks (%)	1 (4)
35–37 weeks (%)	2 (8)
>37 weeks (%)	2 (8)
Weight (range)	885 (491–2950)
<1000 g (%)	14 (56)
1000–1500 g (%)	6 (24)
>1500 g (%)	5 (20)
Comorbidities	
Respiratory distress syndrome (%)	20 (80)
Bronchopulmonary dysplasia (%)	11 (44)
Retinopathy of prematurity (%)	7 (28)
Necrotizing enterocolitis (%)	3 (12)
Intraventricular hemorrhage (%)	1 (4)

**Table 3:** The description of central catheter insertion included dwell time, days between catheter insertion and the onset of sepsis, and management after the first positive blood culture (removal versus retainment). ECC = epicutaneo-caval catheter, CICC = centrally inserted central catheter, UVC = umbilical venous catheter.

Type of Catheter	ECC	CICC	UVC
Total no. (%)	22 (88)	2 (8)	1 (4)
Median dwell time (days, range)	15 (6–21)	13 (11–14)	6
Mean days between insertion and sepsis	9	8	5
Removed (%) *	15 (68)	1 (50)	1 (100)
Retained (%) *	7 (32)	1 (50)	0 (0)

* Percentage of the total number of each catheter group.

**Table 4:** Sample antibiogram of *S. capitis* strains isolated from our population. R = resistant, S = sensitive, MIC = minimum inhibitory concentration.

Antibiotic	MIC	
Clindamycin	0.25	S
Clindamycin-Induced Resistance	Neg	
Gentamicin	8	R
Oxacillin	>2	R
Trimethoprim/Sulfamethoxazole	≤10	S
Vancomycin	1	S

## Data Availability

The data presented in this study are available upon request from the corresponding author. The data are not publicly available due to privacy concerns.
